# Induction of apoptosis in imatinib sensitive and resistant chronic myeloid leukemia cells by efficient disruption of bcr-abl oncogene with zinc finger nucleases

**DOI:** 10.1186/s13046-018-0732-4

**Published:** 2018-03-20

**Authors:** Ningshu Huang, Zhenglan Huang, Miao Gao, Zhenhong Luo, Fangzhu Zhou, Lin Liu, Qing Xiao, Xin Wang, Wenli Feng

**Affiliations:** 1 Department of Clinical Hematology, Key Laboratory of Laboratory Medical Diagnostics Designated by the Ministry of Education, Chongqing Medical University, No.1, Yixueyuan Road, Chongqing, 400016 People’s Republic of China; 2 Department of Laboratory Medicine, The First Affiliated Hospital of Chongqing Medical University, Chongqing, 400016 People’s Republic of China; 3 Department of Hematology, The First Affiliated Hospital, Chongqing Medical University, Chongqing, 400016 People’s Republic of China

**Keywords:** Chronic myeloid leukemia, Bcr-abl, Zinc finger nucleases, Homology-directed repair, Oncogenicity

## Abstract

**Background:**

The bcr-abl fusion gene is the pathological origin of chronic myeloid leukemia (CML) and plays a critical role in the resistance of imatinib. Thus, bcr-abl disruption-based novel therapeutic strategy may warrant exploration. In our study, we were surprised to find that the characteristics of bcr-abl sequences met the design requirements of zinc finger nucleases (ZFNs).

**Methods:**

We constructed the ZFNs targeting bcr-abl with high specificity through simple modular assembly approach. Western blotting was conducted to detect the expression of BCR-ABL and phosphorylation of its downstream STAT5, ERK and CRKL in CML cells. CCK8 assay, colony-forming assay and flow cytometry (FCM) were used to evaluate the effect of the ZFNs on the viablity and apoptosis of CML cells and CML CD34^+^ cells. Moreover, mice model was used to determine the ability of ZFNs in disrupting the leukemogenesis of bcr-abl in vivo.

**Results:**

The ZFNs skillfully mediated 8-base *Not*I enzyme cutting site addition in bcr-abl gene of imatinib sensitive and resistant CML cells by homology-directed repair (HDR), which led to a stop codon and terminated the translation of BCR-ABL protein. As expected, the disruption of bcr-abl gene induced cell apoptosis and inhibited cell proliferation. Notably, we obtained similar result in CD34^+^ cells from CML patients. Moreover, the ZFNs significantly reduced the oncogenicity of CML cells in mice.

**Conclusion:**

These results reveal that the bcr-abl gene disruption based on ZFNs may provide a treatment choice for imatinib resistant or intolerant CML patients.

**Electronic supplementary material:**

The online version of this article (10.1186/s13046-018-0732-4) contains supplementary material, which is available to authorized users.

## Background

The bcr-abl oncogene, a fusion of bcr and c-abl sequences, results from the t(9;22)(q34;q11) chromosomal translocation and encodes BCR-ABL fusion protein with elevated tyrosine kinase activity [1–4]. As a oncogenic kinase, BCR-ABL abnormally activates several downstream signaling pathways including RAS-MAPK, STAT5 and CRKL, which contribute to inhibition of apoptosis and induction of malignant transformation [5]. The first-generation tyrosine kinase inhibitor (TKI) imatinib, as the first-line treatment of CML, inhibits the phosphorylation of BCR-ABL and the activation of multiple downstream substrates [1, 6]. However, more than 25% of CML patients develop imatinib intolerance or resistance and ~ 50% of these patients harbor bcr-abl point mutations [7, 8]. The second and third generation of TKIs have different sensitivities and potencies to the mutations [6, 9], but these drugs require long-term medication which enhance economic burden and adverse drug events [8, 10–13]. Consequently, the resistance to TKIs is still the primary problem need to be solved in CML treatment.

Ultimately, the bcr-abl gene is the underlying cause of the CML pathogenesis and TKI-resistance. Therefore, in theory, a method to destroy bcr-abl gene will fundamentally solve the problem of CML onset and drug resistance. The technique known as ‘genome editing’ such as zinc finger nucleases (ZFNs) [14–16], transcription activatorlike effector nucleases (TALENs) [17–19] and clustered regulatory interspaced short palindromic repeats (CRISPR) / Cas9 [20, 21] is opening the possibility of disrupting bcr-abl oncogene. Analysis of the bcr-abl sequence analysis shows that it is ideal for the construction of ZFNs. ZFNs are generated by fusing the sequence-specific DNA-binding domain to endonuclease domain of *Fok*I [22, 23]. DNA-binding domain is composed of C2H2 zinc-finger proteins (ZFPs). Each finger recognizes 3-base pairs of DNA [24, 25] and has the highest affinity to the 5’-GNN-3′ nucleotide triplet [26]. Three to four zinc-finger domains linked together in tandem to constitute each of ZFN dimers and bind to 18-bp to 24-bp targeting DNA, such a long site is rarer cleavage targets even in complex genomes [27]. In the coding sequence of bcr-abl, there are 15 sites containing the best binding 5’-GNN-3′ sequence fit to generate ‘three fingers ZFNs’ and among these sites even have 3 sites suitable to construct ‘four fingers ZFNs’. The design of ZFNs aim at these sites can effectively and specifically modify bcr-abl and also reduce the off-target cleavage. Therefore, ZFNs are the preferred technology for bcr-abl gene editing. *Fok*I domain, a nonspecific restriction enzyme, cuts the DNA sequence identified by the ZFPs and introduces a DNA double strand break (DSB) [22]. Off-target effects and cellular toxicity by ZFNs can be induced by the homodimers formation of wild-type *Fok*I [27–29]. To address this problem, *Fok*I nuclease variants have been used to eliminate the unwanted homodimers and cleave DNA only as a heterodimer pair [28–30].

The lesion of DSB by ZFNs can be repaired by non-homologous end joining (NHEJ) or homology-directed repair (HDR) [27, 31]. NHEJ is a process that the lesion can be repaired by directly ligating the two broken ends of DSB which does not need a repair template. It can be accurate but repairing DSB with nucleotide mismatches eventually results in insertion or deletion mutations around the break site [32]. However, when a homologous donor DNA template is provided with ZFNs, the rate of HDR at the lesion observably increase [27, 33, 34]. HDR transfers information from homologous donor DNA to the breaks which can achieve introducing precise changes to defined genomic sequences [35]. Following this principle, HDR allows researchers to take advantage of a suitably designed exogenous DNA template to alter or replace the mutated gene directly.

Considering the significant role of bcr-abl in the pathogenesis of CML and resistance of TKI, and importantly, the sequence of bcr-abl is highly suitable for ZFNs construction, we designed ZFNs to targeted disrupt the bcr-abl gene by modular assembly. The modular assembly is the easiest and high-efficiently designed approach for making active ZFNs [36] and has designed a number of active ZFNs to modify the endogenous gene in higher eukaryotic cells [37]. This method generates candidate ZFPs based on identifying fingers to bind a component triplet and these fingers are then linked to target the corresponding sequence. As we know, the bcr-abl containing the first exon of bcr which includes a coiled-coil domain, Tyr177, SH2 binding domain and a serine/threonine kinase domain, is crucial to induce chronic-phase CML [36, 38–41]. Moreover, we found that the sequences of bcr exon1 containing the 5’-GNNGNNGNNGNN-3′ consensus sequence which fit the characteristics of creating an active ZFNs by modular assembly [26]. When the ZFNs targeting the exon1 of bcr-abl and the donor DNA sequence containing a *Not*I enzyme cutting site composed of 8-base were co-delivered into cells, these 8-base were integrated into the exon1 of bcr-abl sequence by HDR and generated a stop codon in the downstream of ZFNs cleavage site, ultimately leading to premature termination of BCR-ABL translation. In view of the above findings, we designed a bcr-abl gene editing approach based on ZFNs. Here, we investigated whether our ZFNs can effectively cut the bcr-abl gene and decrease the expression of BCR-ABL and its downstream molecules. In addition, we evaluated the effect of our ZFNs on inhibiting the malignant proliferation and inducing apoptosis of CML CD34^+^ cell. Importantly, in vivo experiments were made to determine whether the bcr-abl oncogenicity was also destructed by the ZFNs.

## Methods

### Cell lines and cell culture

K562 (Cell Bank of Shanghai Institute of Cell Biology, Chinese Academy of Sciences) and K562/G01 cells were maintained in RPMI 1640 (Gibco, USA) containing 10% fetal bovine serum (Gibco, USA). The resistant cell line, K562/G01, was screened from K562 by culturing with successively increased concentrations of imatinib for several months [42]. For 32D cells, additional 1 ng/ml of murine IL-3 (PeproTech, USA) was supplemented. 293 T and HepG2 cells were cultured in Dulbecco’s modified Eagle medium (DMEM) contained of 10% fetal bovine serum. All cells were cultured at 37 °C in a 5% CO_2_ humidified incubator.

### Nucleofection

ZFNs were transfeced using the Amaxa Nucleofector II device together with the cell line nucleofector kit V or human CD34^+^ cells nucleofector kit (Lonza, Basel, Switzerland). 1 × 10^6^ cells were collected, resuspended in 100 μl of the pre-mixed nucleofector solution with DNA plasmids and nucleofected with the program T-016 for K562 and K562/G01, E-032 for 32D, and U-008 for CD34^+^ cells. After nucleofection, the cells were immediatly resuspended in 500 μl pre-warmed medium and maintained in a 12-well plate.

### Constructs

ZFP-L and ZFP-R were designed and assembled as described. *Fok*I plasmids, containing the Sharkey mutations and the ELD/KKR obligate heterodimer mutations, were obtained from Addgene (*Fok*I-L: plasmid #37198; *Fok*I-R: plasmid #37199) containing the Sharkey mutations and the ELD/KKR obligate heterodimer mutations. Assembled ZFNs were cloned into pAdTrack-CMV termed ZFN-L and ZFN-R. FLAG tag was added to the N-terminal of pAdTrack-CMV. The homology arms in the donor, containing left arm and right arm, were amplified through PCR from human and mouse genomic DNA. The left arm (sense: 5’-CGGGGTACCCAGCGATGGGGCTTCCGGCG-3′, antisense: 5’-AAGGAAAAAAGCGGCCGCGGGTTCAACTCGGCGTCCTCGTAGTCG-3′) and right arm (sense: 5’-AAGGAAAAAAGCGGCCGCCCGCTTCCTGAAGGACAACCTGATCG-3′, antisense: 5’-GCTCTAGAGCCAGGATTCCCGACAGGACCCATTTTC-3′) were inserted into pAdTrack-CMV vector at *Kpn*I, *Not*I, and *Xba*I site to generate the donor plasmid.

The amino acid sequences of ZFP-L and ZFP-R were as follows respectively:

ZFP-L: 5’ LEPGEKPYKCPECGKSFSDCRDLARHQRTHTGEKPYKCPECGKSFSDPGNLVRHQRTHTGEKPYKCPECGKSFSDPGALVRHQRTHTGEKPYKCPECGKSFSDCRDLARHQRTHTGKKTS 3’.

ZFP-R: 5’ LEPGEKPYKCPECGKSFSRSDNLVRHQRTHTGEKPYKCPECGKSFSDCRDLARHQRTHTGEKPYKCPECGKSFSDPGNLVRHQRTHTGEKPYKCPECGKSFSRSDNLVRHQRTHTGKKTS 3’.

The binding sites of ZFN-L and ZFN-R to bcr-abl were as follows respectively: 5′- GGCGTCGACGGCGAGGACGCCGAG-3′.

5’-CTCGGCGTCCTCGCCGTCGACGCC-3’.

The cleavage site of ZFNs is 5’-GACTAC-3′.

### T7E1 assay

Genomic DNA was extracted from the cells treated with ZFNs using the Hipure Tissue DNA Mini Kit (Magen, China). PCR amplification of the region surrounding the ZFNs target site was performed using the PrimeSTAR HS DNA Polymeras (TaKaRa) and 100 ng of genomic DNA as template with primer 5’-GACGCCGAGAAGCCCTTC-3’and 5’-AATCCTCAAAACTCCGGGGG -3′. The PCR products were melted and annealed to form heteroduplex DNA. The annealed DNA was treated with T7 endonuclease 1 (New England BioLabs) for 15 min at 37 °C [37]. Data was analyzed by agarose gel electrophoresis. Ratio of cleaved to uncleaved products was calculated as a measure of frequency of gene disruption and the mutation was also analyzed using next-generation sequencing, as described [43].

### Detection of HDR events

K562 cells were transfected with ZFN-L/R and donor vector. After treated for 48 h, the genomic DNA of cells was extracted and then amplified by PCR as described above. Donor DNA was characterized by the *Not*I site they carried, so the PCR products were analyzed by *Not*I enzyme digestion with the following reaction system:1 μl *Not*I, 2 μl 10 × H Buffer, 2 μl 0.1%BSA, 2 μl 0.1% Triton X-100, 1 μg DNA and ddH_2_O up to 20 μl at 37 °C. These results were measured by agarose gel electrophoresis.

### Western blotting

Western blotting assay was performed as previously described [44]. The primary antibodies were as follows: anti-BCR-ABL, anti-Phospho-BCR-ABL, anti-c-Abl, anti-Phospho-STAT5, anti-STAT5, anti-ERK 1/2, anti-Caspase-3 and anti-PARP were all purchased from Cell Signaling Technology (USA), used at 1:1000 dilution. Anti-FLAG monoclonal antibody (Sigma, USA) was added at a concentration of 1:500 and anti-β-Actin (Zhong Shan Jin Qiao, China) antibody was used at 1:1000 dilution. The expression quantity of each protein was normalized against the β-Actin protein expression using image software.

### CCK-8 assay

The treated cells were plated into 96-well plates at a density of 2000 cells per well with 100 μl RPMI 1640 containing 15% fetal bovine serum and cultured at 37 °C in a 5% CO_2_ humidified incubator. To prevent the medium evaporation, we added 100 μl PBS to the wells surrounding the sample wells. At indicated time 10 μl of CCK-8 (Solarbo,China) was added to each well then incubated at 37 °C for 3 h. Then the absorbance at 450 nm was measured by micro-plate reader (Eon, BioTeck, USA). Each assay was repeated for five times.

### Colony-forming assay

Treated cells were collected and plated (300 cells/well) in 24-well plates with methylcellulose for assessing the colony-forming ability. The number of colonies were counted at 7–14 days later, using an inverted microscope. The colony formation assay were performed for five times.

### Immunofluorescence assay

Cells were collected for immunofluoresence assay, washed 3 times by PBS and coated on slides. The cells were fixed in 4% paraformaldehyde, permeabilized with 0.1% Triton X-100 at 37 °C, blocked in 1% BSA with 5% goat serum and incubated primary antibody at 4°C overnight. Next, the cells were incubated with fluorochrome-conjugated secondary antibody (1:200, Zhong Shan Jin Qiao, China) in a dark room at 37 °C for 1 h. Lastly, the nucleus was stained with DAPI.

### Samples and hematopoietic stem cell isolation

Samples were obtained from CML patients, who were initially diagnosed with CML and had not undergone any chemotherapy (Table 1), or anemia individuals from the first affiliated hospital of Chongqing Medical University or the second affiliated hospital of Chongqing Medical University. The CD34^+^ cells were performed using Stemsep human CD34 positive selection cocktail and cultured in StemSpan serum-free expansion medium (Stem Cell Technologies, Canada) supplemented with 50 ng/ml SCF, 10 ng/ml IL-6 and 10 ng/ml IL-3 (PeproTech, USA) at 37 °C in 5% CO_2_. The study was approved by the ethical committee of Chongqing Medical University.

**Table 1:** Patient characteristics

Characteristics	Median (range)	No. of cases
Gender		
Female		1
Male		3
Total		4
Median age, y	43.5y (22-65y)	
Median WBC, ×10^9^/L	216.97 (112.3–399)	
Karyotype		
t (9;22)(q34.1;q11.2)		4

*Abbreviations*: *y* years old, *WBC* white blood cell

### Murine leukemogenesis model

5–6 weeks old female NOD/SCID mice (*n* = 5, each group) were selected and received 250 cGy radiation before injection. 2–4 h later, 5 × 10^6^ K562/G01 cells in 200 μl PBS modified by ZFNs or treated with mock were injected intravenously. The weight change and white blood cells count of mice were monitored weekly. Weight loss, mental fatigue, splenomegaly and leukocyte hyperplasia were considered as the signs and symptoms of CML-like disease in mice.Peripheral blood was collected and incubated with the antibody against human CD45 to analyze the proportion of CD45^+^ cells by flow cytometry. All animal experiments were performed in accordance with the National Institutes of Health guide for the care and use of Laboratory animals (NIH Publications No.8023, revised 1978) and were conducted with the approval of the Biomedical Ethics Committee of Chongqing Medical University.

### Statistical analysis

Statistical analysis was performed using SPSS (Version 17.0) software. All data were expressed as the mean ± SD. Student’s test was used to assess the significant connections among categorical variables. *P* < 0.05 was considered to be statistically significant.

## Results

### Construction of zinc finger nucleases and the homologous template donor DNA

**Fig. 1:**
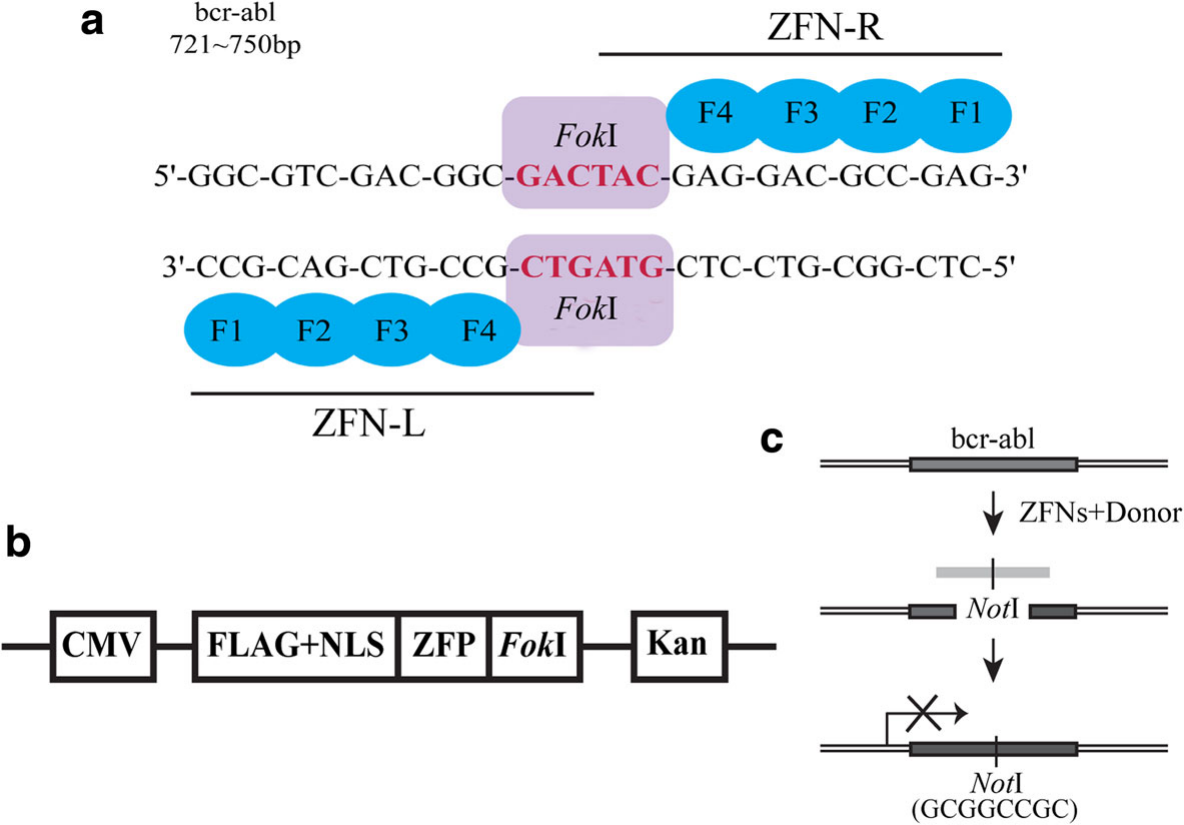
ZFNs were designed to target bcr-abl gene and induce gene modification. **a** Targeted sequence of ZFNs on bcr-abl gene. ZFN designed to cut exon 1 of bcr-abl gene and consisted of four fingers ZFP and a *Fok*I endonuclease. Together the “left hand” (ZFN-L) and “right hand” (ZFN-R) work as dimers to induce a specific DSB. **b** The structure of pAd-Track-ZFN vector. ZFP fused to *Fok*I endonuclease, a nuclear localization signal (NLS) and FLAG tag. The expression of Kanomycin resistance gene (Kan) was regulated by CMV promoter. **c** Sketch of the donor construct and HDR detection scheme. Cleavage of bcr-abl gene created a substrate for HDR, which may use the donor DNA fragment containing a *Not*I site as a repair template. The introduction of *Not*I site, which involved 8-bp, may result in termination of translation

The zinc finger nucleases (ZFNs) targeting exon 1 of the bcr-abl gene, which could cause a double-strand break (DSB), were designed and generated following modular assembly approach [45, 46]. Both of the two zinc finger proteins (ZFPs) (designated ZFP-L and ZFP-R) arrays containing four zinc finger domains were assembled using an archive of ZFP DNA-binding modules [47, 48]. Each of ZFPs was coupled with a codon-optimized *Fok*I domain containing mutations that can prevent homodimer formation and enhance the cleavage activity [30], which is termed as ZFN-L and ZFN-R respectively (Fig. 1a). A nuclear localization signal (NLS) was fused to ZFN and a FLAG tag was incorporated to N-terminal of the protein (Fig. 1b). The NLS allows transportation of ZFN protein to the nucleus binding to the targeted DNA. Our goal is to terminate the translation of BCR-ABL through the direct modification of bcr-abl gene sequence, so we built a suitable donor plasmid to trigger the HDR. The donor sequence containing a *Not*I site, which composed of 8-base, could result in the alteration of the open reading frame and the subsequently premature termination of translation (Fig. 1c).

### ZFN-mediated editing of bcr-abl gene and ending of BCR-ABL protein translation

The applications of gene editing by ZFNs are based on the generation of a site-specific DSB into the interesting sequence. Firstly, we analyzed the expression of ZFNs proteins. The nucleofected K562 cells were harvested at 0 h, 12 h, 24 h, 48 h and 96 h. The result of western blot analysis showed the expression of ZFNs protein can be detected at 12 h after transfection, with a peak at 48 h and diminished at 72 h (Additional file 1: Figure S1A). To demonstrate the nuclear localization of the ZFNs proteins, cells were transfected with ZFN-L and ZFN-R plasmids, together or separately. After 48 h, the amount of ZFNs proteins in the nucleus and cytoplasm were analyzed by western blot respectively. We demonstrated that the engineered ZFNs proteins could localized and expressed in nucleus (Additional file 1: Figure S1B).

**Fig. 2:**
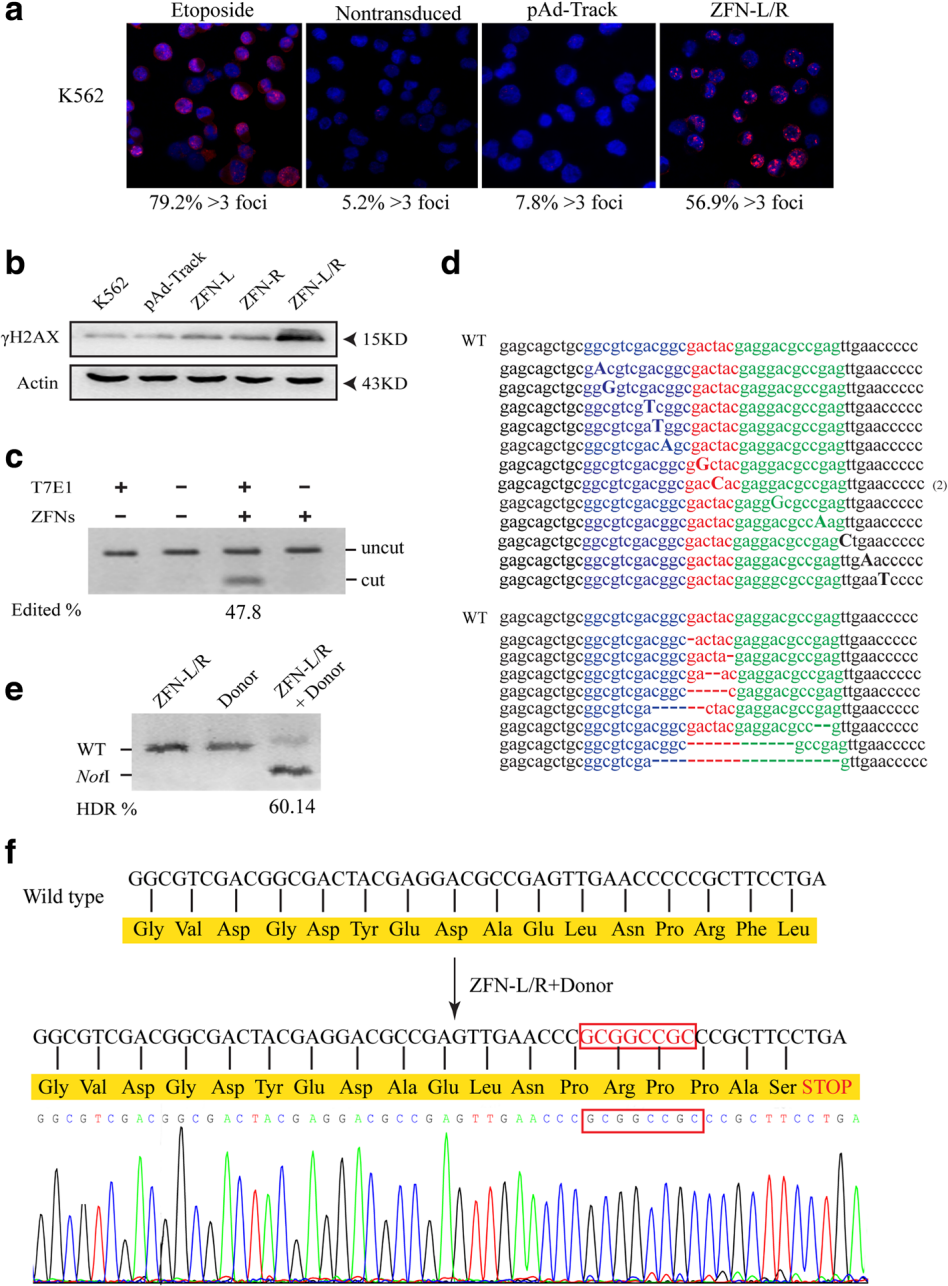
ZFNs induced gene editing of bcr-abl gene. **a** K562 cells were treated with 1 μM etoposide (positive control), pAd-Track or ZFN-L/R for 48 h and ZFNs-induced DSBs were detected by 53BP1 immunostaining. Nontransduced cells as negative control. The rate of cells containing more than 3 foci was shown beneath each panel. **b** K562 cells were transfected with pAd-Track, ZFN-L, ZFN-R plasmids separately or together of ZFN-L and ZFN-R (ZFN-L/R). The amount of γH2AX in each group was quantified by western blot. The arrows indicate the marker proteins. **c** ZFN-mediated gene editing revealed by T7E1 assay and the results indicated by agarose gel eletrophoresis. The bcr-abl was subjected to digestion with T7E1 to confirm the exist of insertions/deletions. Gene modification was only detected in cells transfected with ZFNs shown as ‘cut’ bands. **d** The genomic ZFNs target site in K562 cells was sequenced. The result showed the ZFN-induced insertions and deletions around the target region of bcr-abl. **e** The bcr-abl gene editing efficiency was quantified by *Not*I restriction enzyme. The genomic DNA of cells transfected with ZFN-L/R, Donor individually or together was extracted and amplified by PCR, then treated with *Not*I restriction enzyme. “WT” indicates the position of wild type PCR product and “*Not*I” indicates the position of the fragments generated by *Not*I digestion. Numbers below the lanes with *Not*I fragment indicate the rate of PCR product modification. **f** In silico analysis of sequence of ZFN-L/R and donor treated cells. The result showed the 8-bp (GCGGCCGC) insertion lead to a stop codon and a termination of translation

Next, to determine whether our ZFNs can introduce DSBs at exon 1 of bcr-abl gene, we transfected K562 cells with ZFN-L, ZFN-R separately or together. By detecting the p53-binding protein 1 (53BP1), which forms foci at DNA damage sites, we can evaluate the formation of DSBs by immunofluorescence [28]. Etoposide treated cells as positive control had a high level of 53BP1 foci (79.2% > 3 foci). We observed a low level of 53BP1-stain foci in nontransduced or pAd-Track transduced K562 cells (5.2% > 3 foci and 7.8% > 3 foci respectively) (Fig. 2a). Cells treated with ZFN-L or ZFN-R showed similar level of 53BP1-stain foci (8.3% > 3 foci and 6.7% > 3 foci respectively) (Additional file 2: Figure S2A). In contrast, a dramatic high level of 53BP1 foci (56.9% > 3 foci) was observed in ZFN-L/R group (Fig. 2a). 53BP1 is recruited to the DSBs sites early in the DNA repair response and regarded as the hallmark of DSB. As shown above, ZFN-L/R can, and only can, cause DSBs in K562 cells. To further confirm this result, we analyzed another DNA damage marker, phosphorylated histone H2AX (γH2AX) by western blot. As shown in Fig. 2b, compared with pAd-Track vector and K562 group, the expression of γH2AX was significantly increased in ZFN-L/R group and no significant difference was observed among other conditions (Additional file 2: Figure S2B). The detection of γH2AX expression further confirmed that the DSBs in K562 cells can be induced by ZFN-L/R. Together, these results demonstrate that ZFN-L/R induces DSBs in K562 cells.

Off-target DNA cleavage by ZFNs can be caused by the formation of homodimers between ZFN-L and ZFN-R [28, 29]. To avoid off-target effects, we adopted *Fok*I nuclease variants that are restricted to form heterodimers as described above [45, 49]. To evaluate the activity of ZFNs at the endogenous target sequence, T7 endonuclease I (T7E1) assay was carried out. A ZFNs-induced DSB is typically repaired by NHEJ, which often contains small insertions or deletions (called “indels”) near the break site. The presence of indels in the K562 cells which treated by ZFNs, is confirmed by the mismatch-sensitive T7E1. Then the percentage of bcr-abl gene modification (47.8%) was quantified using gel imaging station (Fig. 2c). Moreover, genomic sequencing verified that the existence of various deletions and insertions resulting from NHEJ repair of the DSBs induced by activity of the ZFNs (Fig. 2d).

To determine whether the ZFNs and donor can mediate BCR-ABL translation termination, K562 cells were transfected with ZFN-L/R and donor, individually or together.

BCR-ABL translation termination, K562 cells were transfected with ZFN-L/R and donor, individually or together. After 48 h, genomic DNA were collected and amplified by semiquantitative PCR followed by digestion with *Not*I restriction enzyme. HDR was observed in 60.14% of the bcr-abl gene (Fig. 2e). Genomic DNA cut by *Not*I was sequenced, results showed a stop codon and an end of translation near the DSB site (Fig. 2f).

### ZFNs targeting bcr-abl depress the expression of BCR-ABL and its downstream signaling pathways

**Fig. 3:**
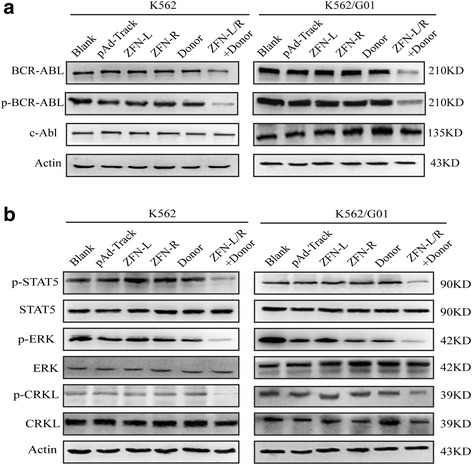
Effect of ZFNs on the expression of BCR-ABL and its downstream signaling pathways. K562 and K562/G01 cells were transfected with pAd-Track, ZFN-L, ZFN-R, Donor or ZFN-L/R and donor. Protein were collected after 48 h for western blotting analysis. **a** Western blot results showed that ZFN-L/R and donor reduced the expression of BCR-ABL and p-BCR-ABL. **b** The activity of p-STAT5, p-ERK and p-CRKL were decreased in the group treated with ZFN-L/R and donor

We performed western blot to confirm whether the ZFNs system can prevent BCR-ABL translation. In K562 and K562/G01 cells, the quantity of BCR-ABL and the activity of p-BCR-ABL in the group treated with donor and ZFN-L/R were significantly lower than that in groups of blank, ZFN-L, ZFN-R or Donor (Fig. 3a, Additional file 3: Figure S3A). We noticed that there was still a few amount of BCR-ABL expression, presumably because the restriction of plasmid transfection efficiency led to a small number of cells did not be edited (in our experiment, the efficiency of transfection of K562 cells can reach 93%. Data not shown). Considering BCR-ABL activates multiple downstream pathways, we further measured key downstream proteins and found that the phosphorylation levels of STAT5, ERK and CRKL were decreased (Fig. 3b, Additional file 3: Figure S3B). Taken together, these results demonstrated that ZFNs targeting bcr-abl caused attenuation of BCR-ABL, accompanied with decreased phosphorylation of its downstream molecules including STAT5, ERK and CRKL.

### ZFNs induce apoptosis and inhibit proliferation of CML cells

**Fig. 4:**
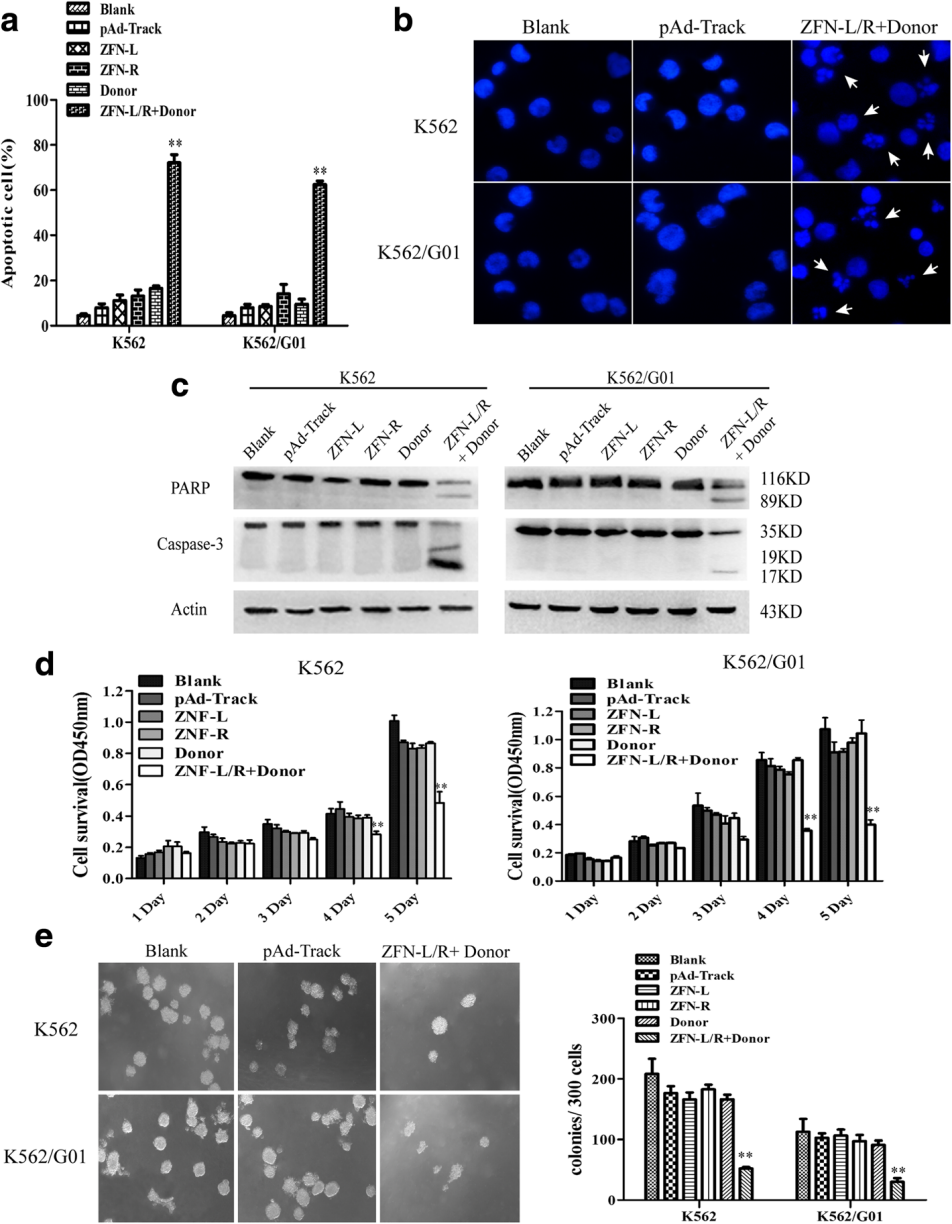
ZFNs induces apoptosis and inhibits proliferation of CML cells. K562 and K562/G01 cells were respectively nucleofected with pAd-Track, ZFN-L, ZFN-R, Donor or ZFN-L/R and donor. **a** The percentage of cell apoptosis was detected by flow cytometry. **b** Morphologic changes of apoptotic cells were detected by DAPI stain. The arrows indicate the prominent apoptotic morphology. **c** Cleavage of PARP and Caspase-3 was detected by western blot. **d** The effect of ZFNs on CML cell proliferation was assessed by CCK-8 assay. **e** Treated cells were plated in 24-well plates with methylcellulose and colonies were counted after 2 weeks. Data are expressed as the means ± SD. **P < 0.01 vs. Controls

To detect the effect of ZFNs on apoptosis of CML cells, we transfected K562 and K562/G01 cells with ZFN-L, ZFN-R, Donor individually or together. Apoptosis was detected by flow cytometry and apoptotic rate significantly increased in donor and ZFN-L/R treated group compared to other groups (Fig. 4a, Additional file 4: Figure S4A). To confirm this effect, we visualized the nuclear morphology changes by DAPI staining. As shown in Fig. 4b, apoptosis characteristic morphological changes, such as condensed and fragmented nuclear, were observed in CML cells treated with ZFN-L/R and donor and no significant changes of nuclear morphology were observed in other groups (Additional file 4: Figure S4B). The presence of BCR-ABL protein makes CML cells resistant to various apoptotic stimuli [50]. Cleaved caspase-3 and PARP as the activation of caspase pathway were measured by western blotting. The result shown that caspase pathway activated in K562 and K562/G01 cells treated with donor and ZFN-L/R (Fig. 4c).

Next, we tested the influence of ZFNs on the proliferation of CML cells. Cell proliferation was measured by CCK-8 assay and colony-forming assay. We found that, since the fourth day, the viability of donor and ZFN-L/R transfected cells was significantly lower than another groups. What’s more, the CML cells in the group treated with donor and ZNF-L/R, from the first day to the fifth day, only had less proliferation (Fig. 4d). The result of colony-forming assay shown that donor and ZNF-L/R co-delivery also inhibited the colony formation ability of CML cells (Fig. 4e, Additional file 5: Figure S5A). Furthermore, to confirm whether the ZFNs system has cytotoxicity on bcr-abl negative cells, we transfected 32D, HepG2 and 293 T cells with the donor and ZFN-L/R expression plasmids and detected the proliferation using CCK-8 assay. The results showed that no significant change was observed in these cells compared with untreated cells (Additional file 5: Figure S5B-D). These results indicated that co-delivery of donor and ZFN-L/R induced apoptosis and suppressed proliferation of imatinib sensitive and resistant CML cells.

### ZFNs induce apoptosis and inhibit proliferation of primary CML leukemia stem/progenitor cells

**Fig. 5:**
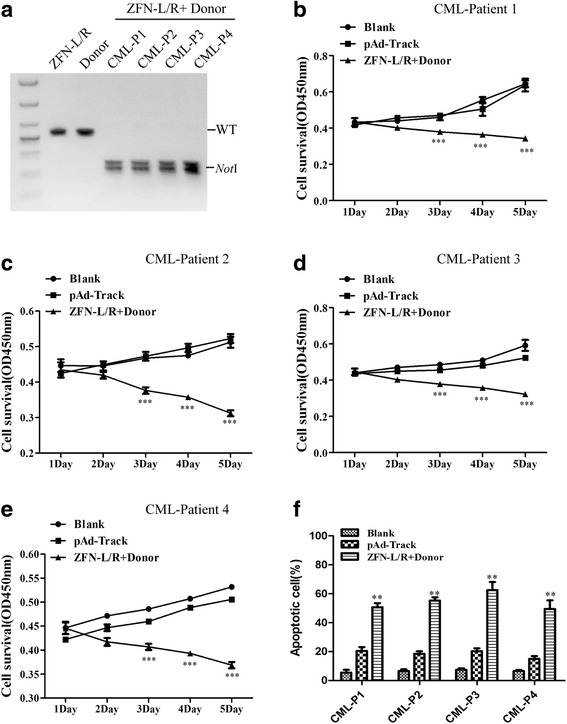
ZFNs induces apoptosis and inhibits proliferation of CD34^+^ cells from CML patients. CML CD34^+^ cells were collected from patients and treated with ZFN-L/R and Donor plasmid, respectively or together. **a** The editing efficiency of ZFNs on bcr-abl gene editing was quantified by *Not*I restriction enzyme. CML-P1, CML-P2, CML-P3 and CML-P4 are abbreviations for CML-Patient 1, CML-Patient 2, CML-Patient 3 and CML-Patient 4 respectively. Cell viability was assessed via CCK8 assay (**b**), (**c**), (**d**), (**e**) and the percentage of apoptotic cells was determined by FCM (**f**). The data are shown as the mean ± SD. **P < 0.01 vs. Controls and ***P < 0.001 vs. Controls

To determine whether our ZFNs have effect on bcr-abl in primary leukemia stem/progenitor cells, we collected CD34^+^ cells from CML patients and transfected them with ZFN-L/R and donor. The efficiency of gene modification was measured by PCR-amplification followed by *Not*I digestion and agarose gel electrophoresis. These results showed that the combination of donor and ZFN-L/R was able to induce gene editing in CD34^+^ cells from CML patients (Fig. 5a). Next, we detected the viability of modified cells by CCK-8 assay and apoptosis through flow cytometry. ZFN-L/R and donor DNA significantly suppressed the proliferation and promoted the apoptosis of CD34^+^ cells from CML patients (Fig. 5b-f), but had no effect on CD34^+^ cells from bcr-abl negative patients who suffered from anemia (Additional file 6: Figure S6A-D).

### ZFNs impair the pathogenicity of bcr-abl in vivo

**Fig. 6:**
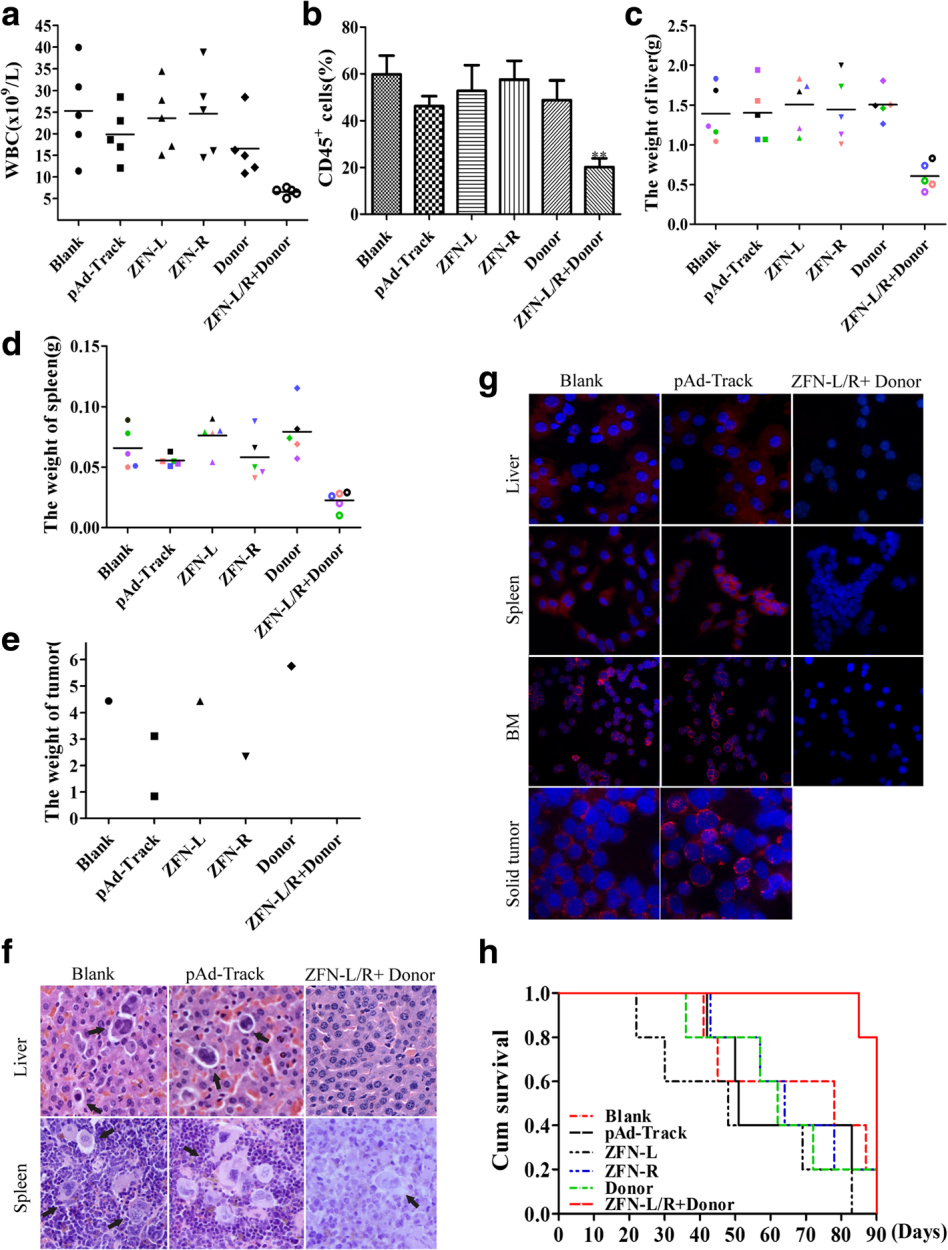
ZFNs impairs the pathogenecity of bcr-abl in mice. **a** The maximum of WBC counts of each mouse in Blank, pAd-Track, ZFN-L, ZFN-R, Donor or ZFN-L/R and donor groups were recorded. **b** The rate of human CD45^+^ cells in murine bone marrow were detected by flow cytometry. **c**, **d** Mean liver or spleen weight of mice in different group was quantified. **e** The weight of solid tumor for each groups were recorded. **f** Infiltration of liver or spleen in groups of Blank, pAd-Trackand or ZFN-L/R + donor was analysed by H&E. The arrows indicate the infiltrating leukemic cells. **g** Detection of BCR-ABL protein by immunofluorescent assay infiltrating. **h** Survival curves were measured by Kaplan-Meier methods

In order to determine the effects of bcr-abl disruption by ZFNs in vivo, K562/G01 cells with treatments or no treatment were injected into NOD/SCID mice through tail vein. The treatments include transfection with pAd-Track, ZFN-L, ZFN-R, Donor or donor with ZFN-L/R. The white blood cell counts of mice in groups were monitored weekly and the peak number of each mouse was recorded. As shown in Fig. 6a, the peak level of leukocytes ZFN-L/R and donor treated mice reached was significantly lower when compared to other groups. To confirm whether these leukocytes were leukemic or not, peripheral blood was collected from each group and CD45 antigen which expressed on the membrane surface of human leukocytes were detected by FCM. The number of CD45 positive cells indicated the number of K562/G01 cells propagating in murine bone marrow. As shown in Fig. 6b, the donor and ZFN-L/R group had lower level of CD45-positive cells than another groups. These results proved that ZFN-L/R and donor suppressed the proliferation of K562/G01 cells in NOD-SCID mice. Furthermore, mice in group blank, ZFN-L, ZFN-R or Donor developed with more severe hepatomegaly and splenomegaly compared with ZFN-L/R and donor group (Fig. 6c-d, Additional file 7: Figure S7A). There was no mice harboring solid tumor in group treated with donor and ZFN-L/R. In contrast, in another groups there was one to two mice suffered from solid tumor in enterocoelia, buttock or cheek (Fig. 6e, Additional file 7: Figure S7B). Infiltration of leukemic cells in tissues was examined by hematoxylin/eosin (HE) and Wright’s staining, and the BCR-ABL expression was detected via immunofluorescent. Mice treated with ZFN-L/R and donor were observed with less leukemic cell infiltration in the liver and spleen (Fig. 6f, Additional file 7: Figure S7C). By Wright’s staining of bone marrow, a reduced Myeloid: Erythroid ratio was shown in ZFN-L/R and donor group when compared with other groups (Additional file 7: Figure S7D). Moreover, a high level expression of BCR-ABL was detected in cells from livers, spleens, bone marrow or soild tumors from blank, ZFN-L, ZFN-R or Donor treated mice (Fig. 6g or not shown). Kaplan-Meier survival analysis showed that the mice receiving ZFN-L/R and donor treated cells demonstrated significantly longer survival time than another groups (Fig. 6h). In summary, co-delivery of ZFN-L/R and donor was able to inhibit the proliferation and infiltration of K562/G01 cells, ultimately, delay the onset of CML in mice.

## Discussion

CML is a myeloproliferative neoplasm characterized by the t(9;22)(q34;q11) reciprocal chromosomal translocation that generates the bcr-abl fusion gene encoding a constitutive kinase activity which is necessary for CML pathogenesis [51]. Imatinib has revolutionized CML therapy and remains the gold standard for the treatment, but more than 25% of patients will have drug resistance or intolerance [36]. The main cause of resistance to imatinib is the mutations in ABL kinase domain which impair the imatinib binding. The second-generation TKIs, such as nilotinib and dasatinib, retain inhibitory activity against the most of the mutations except the T315I “gatekeeper” mutation [52]. Although the third-generation TKI ponatinib exhibits inhibitory activity against all single mutations, its clinical use is limited by the severe side effects [13, 53, 54]. Besides, TKIs cannot eradicate leukemic stem cells (LSCs) of CML patients which would result in TKI-resistance or relapse [55–57]. Thus, the battle to effectively disrupt the pathological root of CML has been a long fight. In this study, we report a new strategy based on ZFNs editing bcr-abl gene at the protein-coding sequence and terminating the translation of BCR-ABL to destroy the pathogenicity of imatinib sensitive and resistant CML cells in vivo and in vitro.

A successful application of ZFNs contains two important elements: specificity and efficiency. Firstly, high specificity depends on the two components of ZFN. With the progression over decades, the ZFP domain has developed into the engineered peptide being able to bind to almost any DNA sequence [24, 58, 59]. This region consists of C_2_H_2_-zinc fingers, each recognizing 3-bp of DNA. In general, three zinc fingers constitute the individual ZFNs to bind 9-bp targeting DNA. However, the ‘three-finger’ ZFNs had been confirmed with little activity and specificity [30]. Recent studies showed that more fingers of ZFNs (up to six per ZFN) can improved the specificity [33, 60, 61]. In our research, the ZFP designed with four fingers can recognize 12-bp DNA site and such a long site is rarer cleavage targets even in complex genomes. *Fok*I dimerization is another important feature of ZFNs that only dimerized *Fok*I domains can cleave DNA [62]. Moreover, to improve ZFNs specificity, we introduced the codon-optimized *Fok*I domain which cleaves DNA only as a heterodimer pair. The 53BP1 was measured by immunostaining to monitor editing specificity of the ZFNs. We found that DSBs occurred highly above background only in K562 cells co-delivered with ZFN-L and ZFN-R (Fig. 2a-b).

Secondly, the efficiency of genome editing may be controlled by the DSB repair approach. NHEJ is more active than HDR in most of the cell cycle, which makes gene correction and insertion get more challenges [32]. However, when a homology donor DNA is delivered with ZFNs, the rate of HDR is dramatically enhanced at the DSB sites [27, 33, 34]. Based on this principle, ZFN-donor combination has been adopted to achieve efficient gene modification in various mammalian cells [27, 63–67] and also applied to various diseases treatment [68, 69]. As we know, DSB repaired by NHEJ, an error-prone repair pathway, frequently inducing nucleotide indels in break site may lead to gene knockout. Nevertheless, the gene editing achieved by NHEJ is unpredictable and not all the indels are expected. In conclusion, co-delivery of ZFNs and donor can achieve efficient and user-designed gene editing by HDR repair pathway. Therefore, in this study, we tried to use HDR-driven gene modification for CML treatment.

To achieve therapeutic editing in clinical, the ZFNs need to be delivered to target cells, which can be performed either in vivo or ex vivo. In vivo genome editing therapy, the ZFNs are delivered directly to diseased cells in the body. This mode of therapy may have the potential to treat diseases that have effects on multiple organ systems. On the other hand, the appropriate vector [70–72], immune response [73] and unpredictable off-target [74] are the potential barriers for application of this therapy mode. In ex vivo therapy the target cells are removed from the body and transfused back into the host after modified with ZFNs. CML is suitable for ex vivo therapy with ZFNs. First, cells of hematopoietic system can survive under manipulated conditions outside the body. Second, in this study, our ZFNs system was capable of efficiently prevent the tumorigenesis potential of BCR-ABL (Figs. 3, 4, 5). For ex vivo application, electroporation can be used to deliver plasmids of ZFNs into hematopoietic stem cells [75]. However, lentiviral vectors which may drive constitutive expression and more off-target activity are less desirable [32].

This is the first time to report a bcr-abl gene disruption approach based on ZFNs which may provide a novel therapeutic strategy for imatinib resistant or intolerant CML patients. The results of CCK-8 assay, colony-forming assay and flow cytometry shown that the ZFNs had potent anti-leukemia ability in vitro. Also, we found that the ZFNs can impaired leukemogenesis of K562/G01 cells in mice by specifically targeting and disrupting bcr-abl gene. As we known, the point mutations in ABL kinase domain were responsible for TKIs resistance in CML patients [76], with this reason considered, we constructed ZFNs targeting BCR domain which also made the ZFNs suitable for treating drug-resistant patients with different mutations. In summary, the ZFNs technology may be the potential and effective method to treat CML.

## Conclusions

The combination of donor and ZFNs demonstrated here is able to truncate BCR-ABL oncoprotein efficiently in CML cells and CML CD34^+^ cells and show the anti-leukemia ability in vitro and in vivo. This approach may provide a promising treatment choice for CML patients, especially for whom with imatinib resistance.

## Additional files

Additional file 1: **Figure S1.** Expression of ZFNs proteins in K562 cells. (A) The proteins of K562 cells were collected after nucleofection of ZFNs at different times (0 to 72 h). Anti-Flag antibody was used to detect the protein expression. (B) K562 cells were transfected with pAd-Track, ZFN-L, ZFN-R plasmids separately or together of ZFN-L and ZFN-R (ZFN-L/R). Nuclear and cytoplasmic proteins were collected, from which the amount of ZFN proteins were detected. The arrows indicate the marker proteins. Data are expressed as the means ± SD. **P < 0.01 vs. Controls. (TIFF 511 kb)

Additional file 2: **Figure S2.** Supporting data for Fig. 2. (A) K562 cells were treated with ZFN-L and ZFN-R for 48 h and ZFNs-induced DSBs detected by 53BP1 immunostaining. The rate of cells containing more than 3 foci was shown beneath each panel. (B) Quantification of γH2AX protein from the experiments, normalized to actin. The data are shown as the mean ± SD. **P < 0.01 vs. Controls. (TIFF 327 kb)

Additional file 3: **Figure S3.** Supporting data for Fig. 3. (A), (B) Quantification of protein from the experiments, normalized to actin. The data are shown as the mean ± SD. **P < 0.01 vs. Controls. (TIFF 745 kb)

Additional file 4: **Figure S4.** Supporting data for Fig. 4. (A) The percentage of apoptotic cells was determined by 7-AAD/AnnexinV-PE staining followed by flow cytometric analysis. (B) K562 and K562/G01 cells were treated with ZFN-L, ZFN-R or Donor. The morphologic changes of apoptotic cells were detected by DAPI stain (TIFF 1504 kb)

Additional file 5: **Figure S5.** Supporting data for Fig. 4. (A) The colony formation ability of K562 and K562/G01 cells which treated with ZFN-L, ZFN-R or Donor. (B), (C), (D) The viability of 32D, HepG2 and 293 T cells treated with pAd-Track or ZFN-L/R and donor was measured by CCK-8 assay. (TIFF 1102 kb)

Additional file 6: **Figure S6.** ZFNs have no effect on the proliferation and apoptotic rate of CD34^+^ cells from anemia patients. CD34^+^ cells were collected from anemia patients and treated with ZFN-L/R and Donor plasmid, respectively or together. (A), (B), (C) Cell viability was assessed via CCK8 assay. (D) The percentage of apoptotic cells was determined by FCM. (TIFF 424 kb)

Additional file 7: **Figure S7.** Supporting data for Fig. 6. (A) Representative images of livers and spleens from groups of Blank, pAd-Track, ZFN-L, ZFN-R, Donor or ZFN-L/R and donor. (B) Comparison of the size of solid tumor from each groups. (C) Infiltration of liver and spleen in group of ZFN-L, ZFN-R and Donor was analysed by H&E. The arrows indicate the infiltrating leukemic cells. (D) Bone marrow cells from mice in each group were stained with Wright’s staining. The arrows indicate the typical leukemic cells. (TIFF 4194 kb)

## Abbreviations

CML: Chronic myeloid leukemia
DSB: Double strand break
FCM: Flow cytometry
HDR: Homology-directed repair
HE: Hematoxylin/eosin
indels: Insertions or deletions
LSCs: Leukemic stem cells
NHEJ: Non-homologous end joining
NLS: Nuclear localization signal
T7E1: T7 endonuclease 1
TKI: Tyrosine kinase inhibitor
WBC: White blood cells
ZFNs: Zinc finger nucleases
ZFPs: Zinc finger proteins
