# Supplementary figures and images for: Induction of apoptosis in imatinib sensitive and resistant chronic myeloid leukemia cells by efficient disruption of bcr-abl oncogene with zinc finger nucleases
Source: J Exp Clin Cancer Res. 2018 Mar 20;37:62. doi: 10.1186/s13046-018-0732-4 (PMC5859405; doi:10.1186/s13046-018-0732-4)

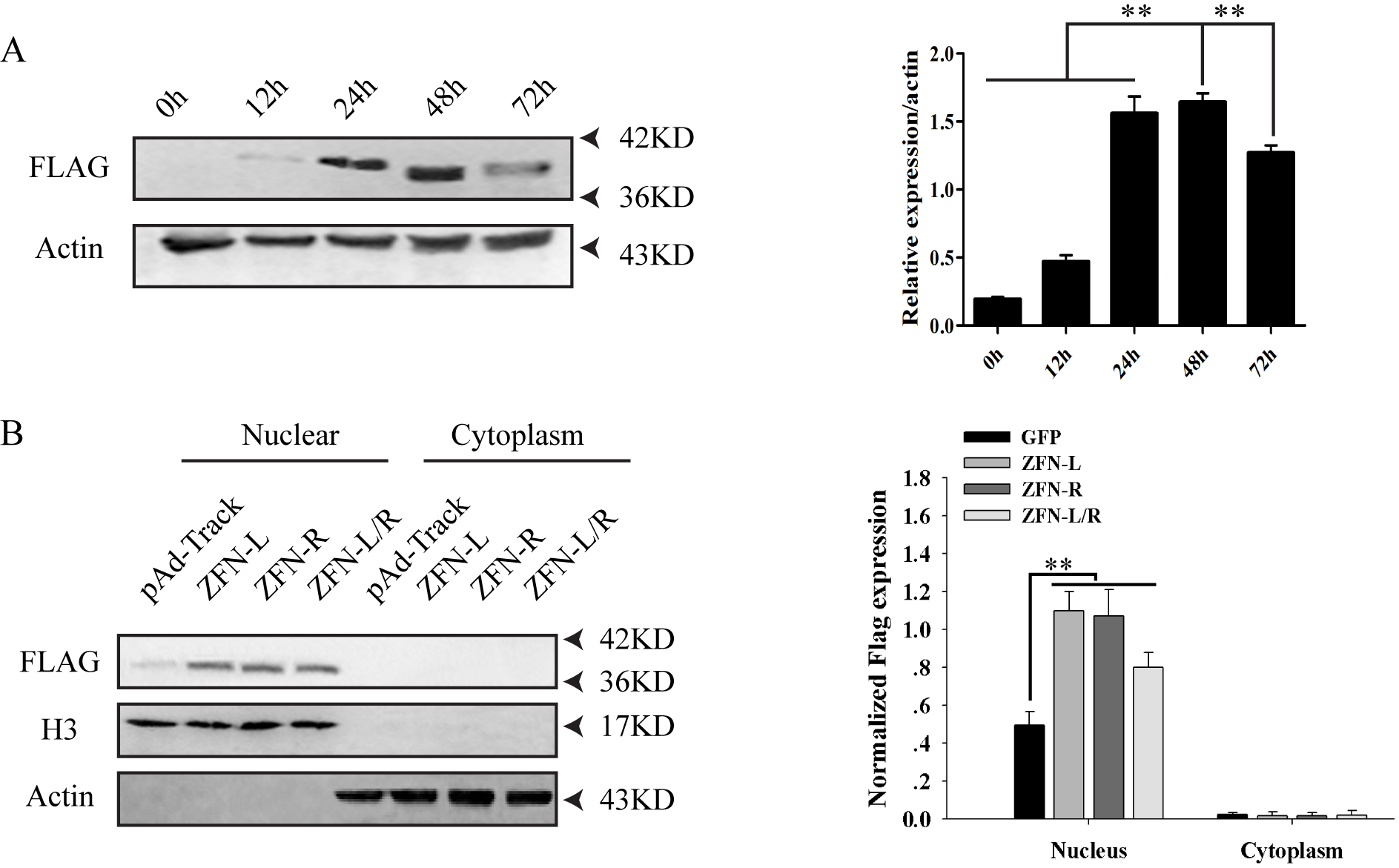

Supplement: Supplementary file 1 — Figure S1. Expression of ZFNs proteins in K562 cells. (A) The proteins of K562 cells were collected after nucleofection of ZFNs at different times (0 to 72 h). Anti-Flag antibody was used to detect the protein expression. (B) K562 cells were transfected with pAd-Track, ZFN-L, ZFN-R plasmids separately or together of ZFN-L and ZFN-R (ZFN-L/R). Nuclear and cytoplasmic proteins were collected, from which the amount of ZFN proteins were detected. The arrows indicate the marker proteins. Data are expressed as the means ± SD. **P < 0.01 vs. Controls. (TIFF 511 kb) [file 13046_2018_732_MOESM1_ESM.tif]

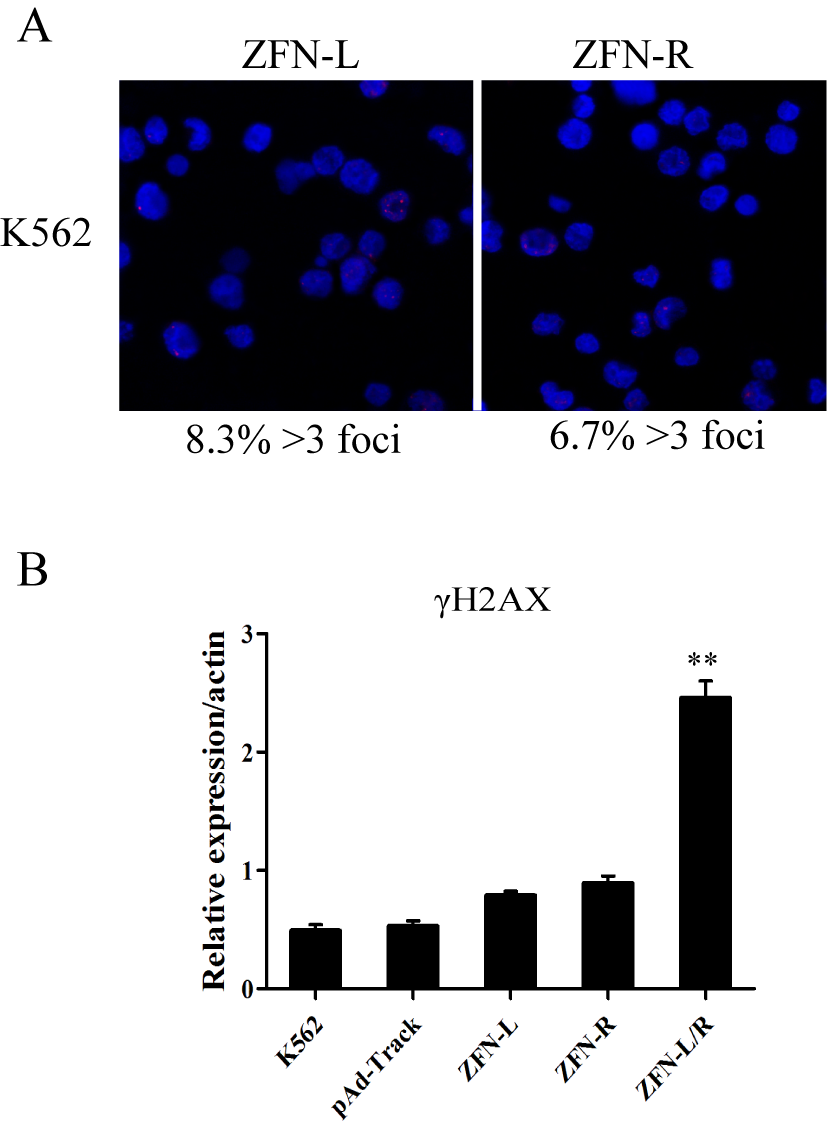

Supplement: Supplementary file 2 — Figure S2. Supporting data for Fig. 2. (A) K562 cells were treated with ZFN-L and ZFN-R for 48 h and ZFNs-induced DSBs detected by 53BP1 immunostaining. The rate of cells containing more than 3 foci was shown beneath each panel. (B) Quantification of γH2AX protein from the experiments, normalized to actin. The data are shown as the mean ± SD. **P < 0.01 vs. Controls. (TIFF 327 kb) [file 13046_2018_732_MOESM2_ESM.tif]

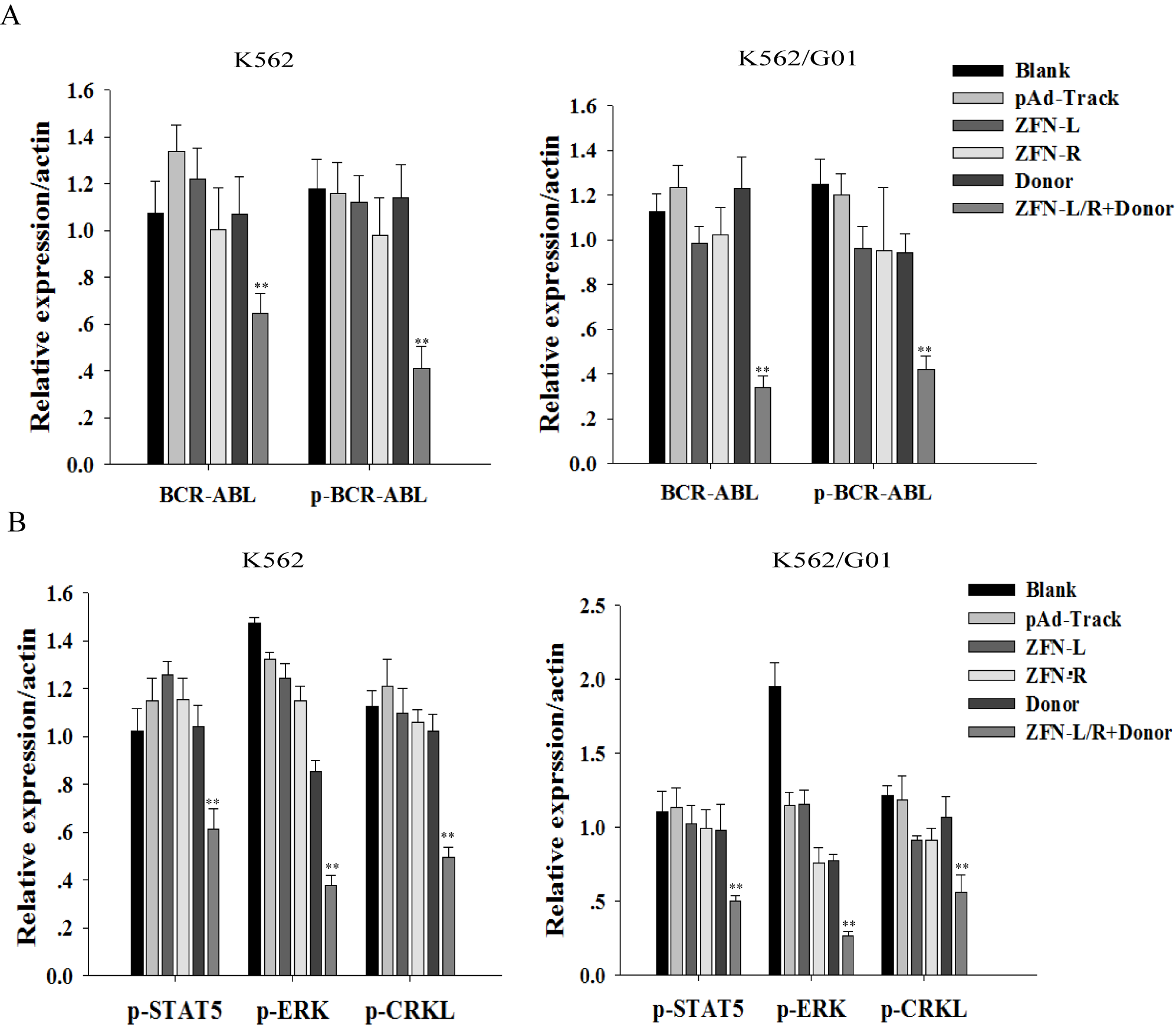

Supplement: Supplementary file 3 — Figure S3. Supporting data for Fig. 3. (A), (B) Quantification of protein from the experiments, normalized to actin. The data are shown as the mean ± SD. **P < 0.01 vs. Controls. (TIFF 745 kb) [file 13046_2018_732_MOESM3_ESM.tif]

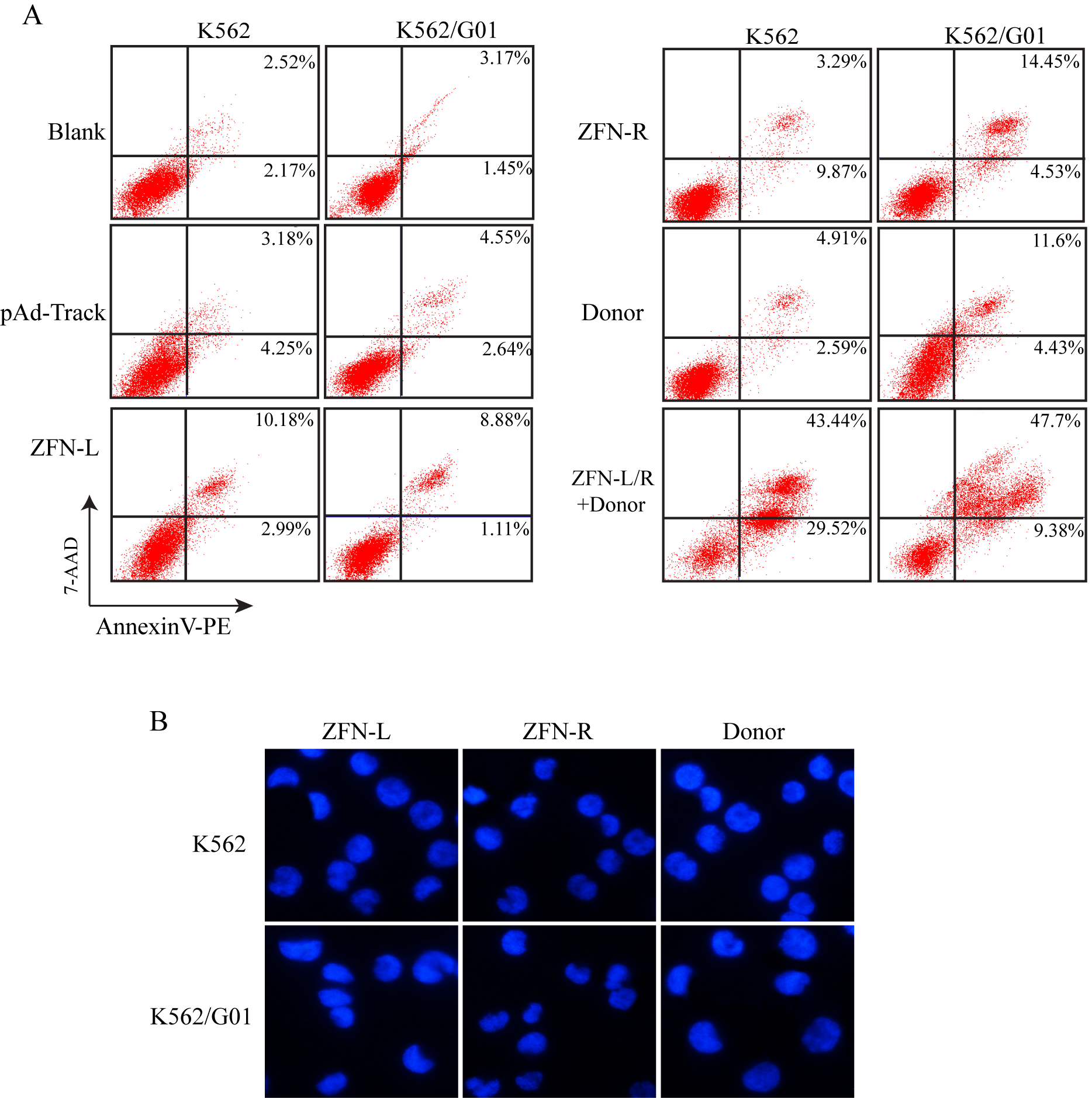

Supplement: Supplementary file 4 — Figure S4. Supporting data for Fig. 4. (A) The percentage of apoptotic cells was determined by 7-AAD/AnnexinV-PE staining followed by flow cytometric analysis. (B) K562 and K562/G01 cells were treated with ZFN-L, ZFN-R or Donor. The morphologic changes of apoptotic cells were detected by DAPI stain (TIFF 1504 kb) [file 13046_2018_732_MOESM4_ESM.tif]

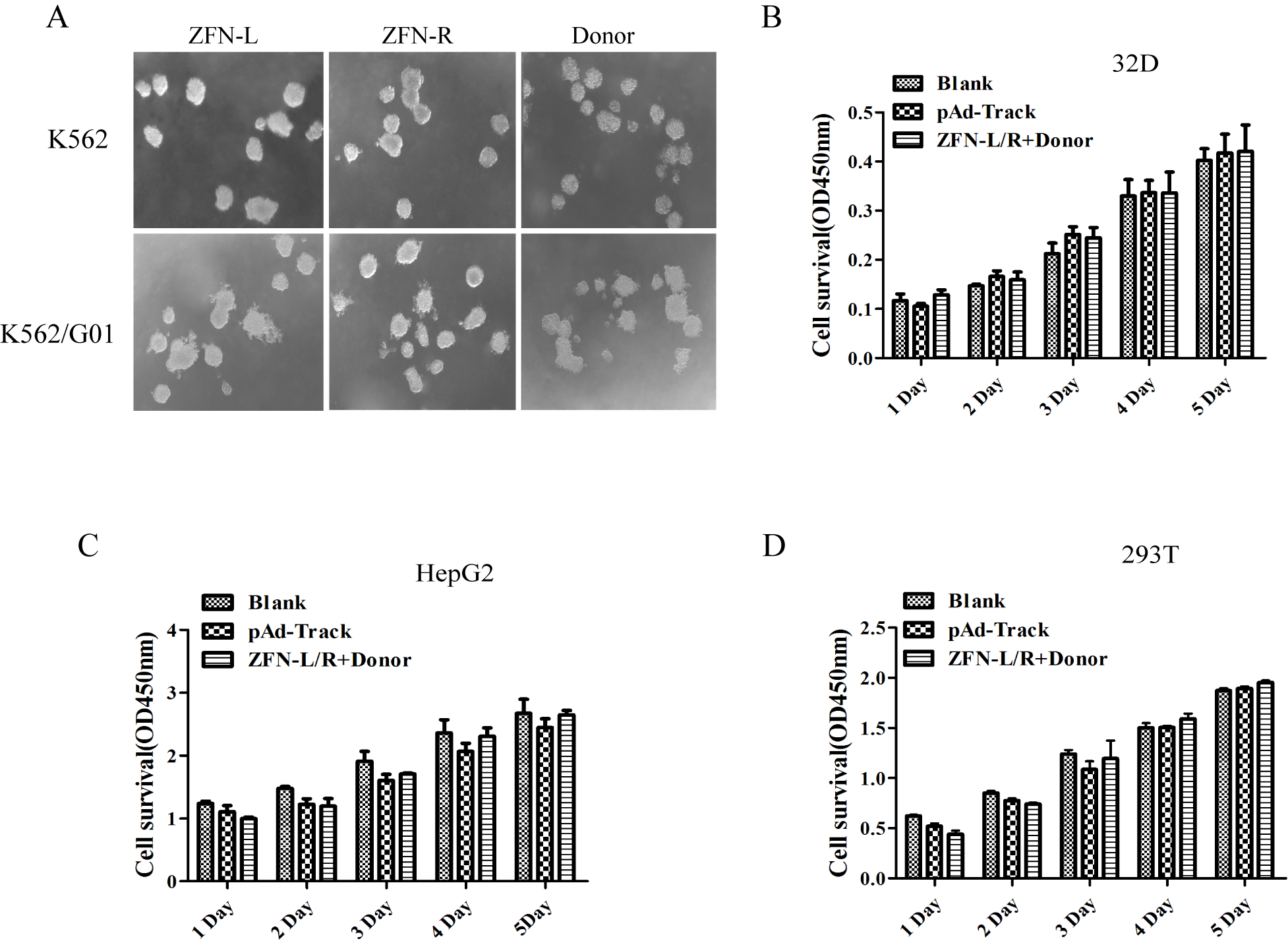

Supplement: Supplementary file 5 — Figure S5. Supporting data for Fig. 4. (A) The colony formation ability of K562 and K562/G01 cells which treated with ZFN-L, ZFN-R or Donor. (B), (C), (D) The viability of 32D, HepG2 and 293 T cells treated with pAd-Track or ZFN-L/R and donor was measured by CCK-8 assay. (TIFF 1102 kb) [file 13046_2018_732_MOESM5_ESM.tif]

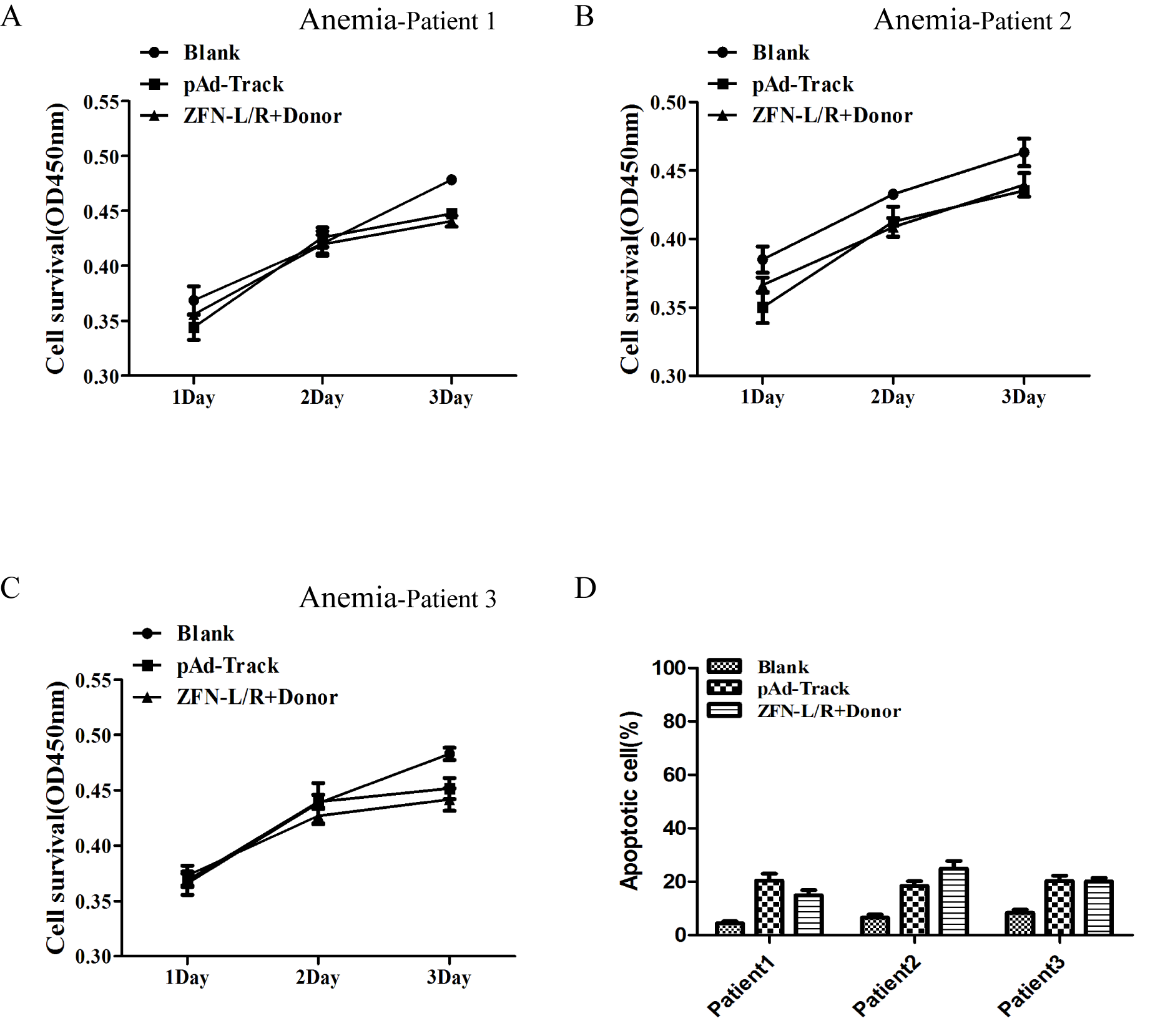

Supplement: Supplementary file 6 — Figure S6. ZFNs have no effect on the proliferation and apoptotic rate of CD34+ cells from anemia patients. CD34+ cells were collected from anemia patients and treated with ZFN-L/R and Donor plasmid, respectively or together. (A), (B), (C) Cell viability was assessed via CCK8 assay. (D) The percentage of apoptotic cells was determined by FCM. (TIFF 424 kb) [file 13046_2018_732_MOESM6_ESM.tif]

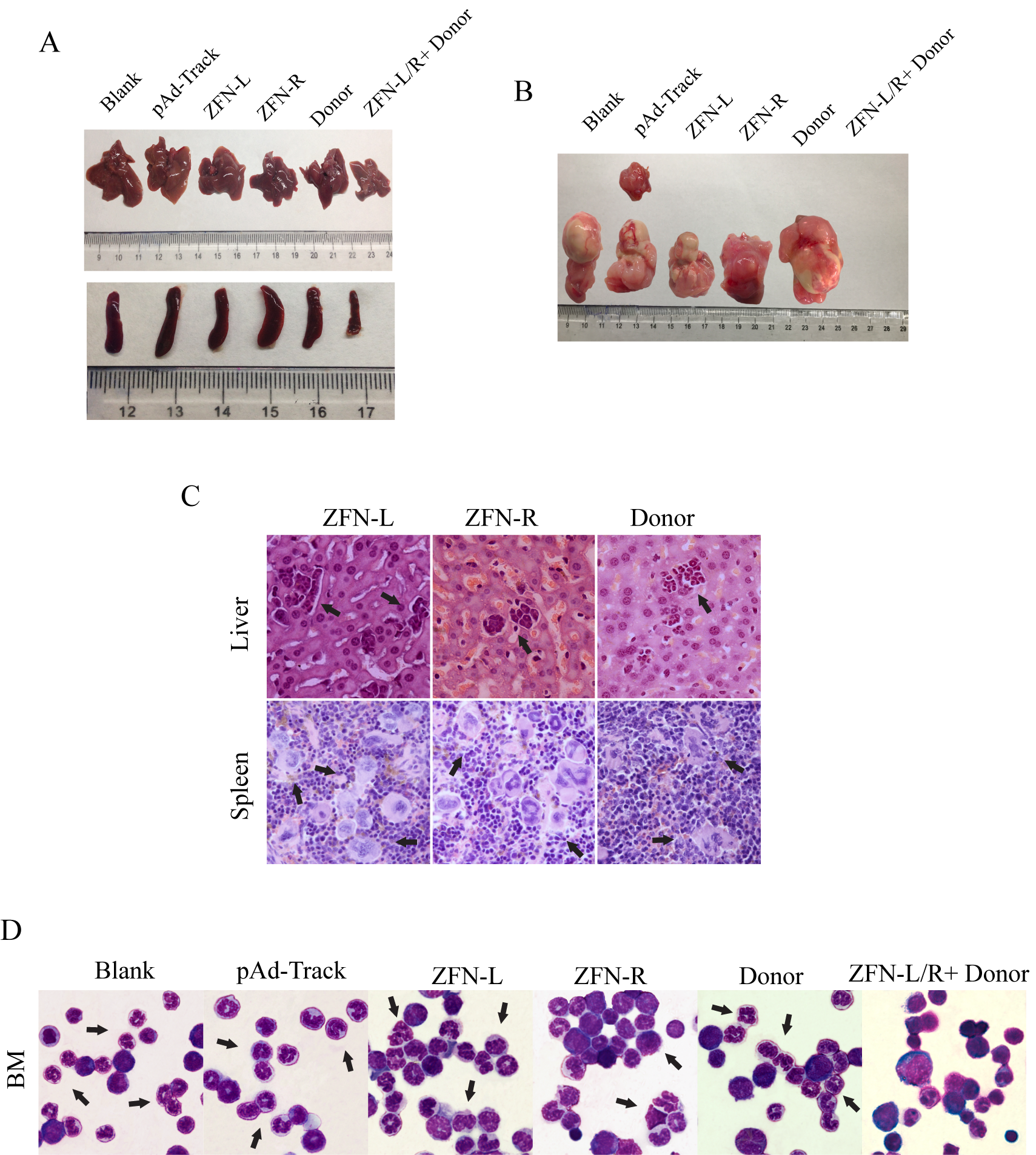

Supplement: Supplementary file 7 — Figure S7. Supporting data for Fig. 6. (A) Representative images of livers and spleens from groups of Blank, pAd-Track, ZFN-L, ZFN-R, Donor or ZFN-L/R and donor. (B) Comparison of the size of solid tumor from each groups. (C) Infiltration of liver and spleen in group of ZFN-L, ZFN-R and Donor was analysed by H&E. The arrows indicate the infiltrating leukemic cells. (D) Bone marrow cells from mice in each group were stained with Wright’s staining. The arrows indicate the typical leukemic cells. (TIFF 4194 kb) [file 13046_2018_732_MOESM7_ESM.tif]
